# Statistical bounds on how induced seismicity stops

**DOI:** 10.1038/s41598-022-05216-9

**Published:** 2022-01-24

**Authors:** Ryan Schultz, William L. Ellsworth, Gregory C. Beroza

**Affiliations:** Department of Geophysics, Stanford University, Stanford, CA USA

**Keywords:** Natural hazards, Seismology, Geophysics

## Abstract

Earthquakes caused by human activities receive scrutiny due to the risks and hazards they pose. Seismicity that occurs after the causative anthropogenic operation stops has been particularly problematic—both because of high-profile cases of damage caused by this trailing seismicity and due to the loss of control for risk management. With this motivation, we undertake a statistical examination of how induced seismicity stops. We borrow the concept of Båth’s law from tectonic aftershock sequences. Båth’s law anticipates the difference between magnitudes in two subsets of seismicity as dependent on their population count ratio. We test this concept for its applicability to induced seismicity, including ~ 80 cases of earthquakes caused by hydraulic fracturing, enhanced geothermal systems, and other fluid-injections with clear operational end points. We find that induced seismicity obeys Båth’s law: both in terms of the magnitude-count-ratio relationship and the power law distribution of residuals. Furthermore, the distribution of count ratios is skewed and heavy-tailed, with most earthquakes occurring during stimulation/injection. We discuss potential models to improve the characterization of these count ratios and propose a Seismogenic Fault Injection Test to measure their parameters in situ. We conclude that Båth’s law quantifies the occurrence of earthquake magnitudes trailing anthropogenic operations.

## Introduction

The injection of fluids in the subsurface has the potential to cause faults to reactivate with earthquake slip^1,2^. Numerous high-magnitude cases of induced seismicity causing significant impacts^3–6^ have led to increased attention. Study topics have covered a broad range of aspects including triggering mechanisms, maximum magnitude, potential risks/hazards, and managements strategies^7,8^. Despite the proliferation of scientific studies, relatively little attention has been devoted to understanding how induced earthquake sequences stop. This is surprising because earthquakes trailing well shut-in have been identified as one of the most significant sources of risk for induced seismicity management^9^. In practice, there are many cases where the largest magnitude earthquake trails the shut-in of the responsible well^5,10–12^ or where abrupt ‘jumps’ in magnitude have occurred^13^.

In this study we build a conceptual framework for statistical bounds on how induced earthquake sequences stop. We start by following the development of Båth’s law for aftershocks of natural earthquakes and adapt it through a set of assumptions and justifications for induced earthquakes. We test the validity of this framework on sequences of induced seismicity and find that in most cases the majority of earthquakes occur during the stimulation/injection periods, rather than trailing well shut-in. As well, the largest events occurring during the stimulation/trailing periods follow Båth’s law. We also discuss the implications of our results and suggest recommendations for in situ tests that could be useful for constraining induced earthquake risks. Finally, we speculate on how improved physical models could be used to build upon our empirical analysis of how induced earthquakes stop.

## Background information on tectonic aftershocks and Båth’s law

Båth’s law is an empirical observation that the average magnitude difference between a mainshock earthquake and its largest aftershock is approximately 1.2 magnitude units^14^. Statistical models of earthquakes^15,16^ have come to interpret Båth’s law as a probabilistic consequence of two more fundamental observations^17–20^. The first being an aftershock law (i.e., Omori’s law), which describes the temporal decay of earthquake rate following a main shock^21–23^.

1 $$n^{o} \left( t \right) = K\left( {t + c} \right)^{ - p}$$

In Omori’s law $n\left( t \right)$ is the time-dependent rate of aftershocks, *K* describes the productivity of the aftershock sequence and strongly influences the total number of aftershocks, *p* is the decay exponent, and *c* is an ad hoc parameter added to ensure the equation doesn’t diverge at the time of the mainshock (i.e., *t* = 0). In this formulation, the value of *p* is bounded such that $p > 1$ for a finite number of earthquakes to occur^24^. The second fundamental observation is the Gutenberg Richter magnitude frequency distribution (GR-MFD), which describes the relative number of small and large earthquakes in a sample^25,26^.

2 $$N = 10^{a} 10^{ - bM}$$

In the GR-MFD *N* is the sample size of the catalogue, *a* is the *a*-value which is related to the productivity of the sequence, and *b* is the *b*-value, which controls the proportionality of big-to-small magnitude earthquakes.

Omori’s law governs the total number of earthquakes that follow a mainshock *N*_*A*_ and the GR-MFD controls the distribution of earthquake magnitudes ‘randomly’ sampled in that population. The combination of these two observations gives the expression for the average magnitude difference (where *N* > *N*_*A*_ > 1).

3 $$\left\langle {{\Delta M}} \right\rangle \approx \frac{1}{b}log_{10} \left( {N/N_{A} } \right)$$

where the total number of earthquakes in a catalogue *N* is an observable and the number of aftershocks *N*_*A*_ can be forecast/estimated from the Omori’s law parameters. Typical estimates of Omori and GR-MFD parameters yields estimates of the average magnitude difference $\left\langle {{\Delta M}} \right\rangle$ that are similar to the empirical observation of ~ 1.2 M^27,28^.

## Deriving a trailing earthquake model for induced seismicity

Following a similar rationale, we derive a trailing earthquake model from the statistical viewpoint of induced earthquakes as a point-process. We first assume that induced earthquake magnitudes are independent and identically distributed random variables that follow the GR-MFD. This assumption is supported by numerous cases that obeying this distribution^2,29,30^, similar to naturally occurring tectonic sequences. From this assumption an expression for the distribution of the maximum magnitude earthquake follows (in the limit of large *N*).

4 $${\text{M}}_{{{{\max}}}} \approx {\text{M}}_{{\text{C}}} + \frac{1}{b}log_{10} \left( N \right) - \frac{1}{b}log_{10} \left( { - ln\left( u \right)} \right)$$

**Figure 1:**
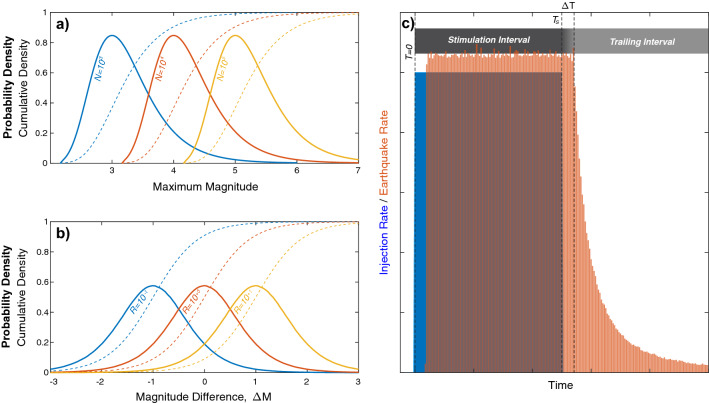
Concepts of Båth’s law and a simple adaptation for trailing seismicity. (**a**) Probability density functions (solid lines) and cumulative density functions (dashed lines) for the expected maximum magnitude for various population sizes *N*, based on numerically sampling Eq. (4). (**b**) Probability density functions (solid lines) and cumulative density functions (dashed lines) for the magnitude difference ΔM for various population ratios *R*, based on numerically sampling Eq. (5). (**c**) Simple model of induced seismicity, where earthquakes lag injection/shut-in by time ΔT and trailing seismicity decays with time.

This is often referred to as the sample size hypothesis^29^ for the distribution of maximum magnitudes M_max_, where the newly introduced parameters are the magnitude of completeness M_C_^31^ and a confidence level $u$ that can be utilized to sample the M_max_ distribution. The distribution of expected maximum magnitudes for different population sizes is shown in Fig. 1a.

Based on this sample size hypothesis, we can consider an archetype of an induced earthquake sequence that is broken into two segments: the injection/stimulation phase (denoted by a subscript S) and the trailing shut-in phase (denoted by a subscript T) that follows (Fig. 1c). The stimulation phase would entail a population count of *N*_*S*_ stimulation earthquakes that occur until the anthropogenic operation is halted at time *T*_*S*_, lagged by stimulation events for time ΔT, and followed by aftershock-like events (starting at an earthquake rate approximately equal to the stimulation rate). We consider trailing seismicity to be those *N*_*T*_ events that occur after time *T*_*S*_ i.e., the sum of lagged stimulation events and the aftershock-like events. Taking the difference of the maximum magnitudes (Eq. 4) for both segments and the stimulation segment yields the relationship for the trailing magnitude difference below.

5 $${\Delta M} = {\text{M}}_{{{\text{max}}}} - {\text{M}}_{{{\text{max}},{\text{S}}}} \approx \frac{1}{b}log_{10} \left( {\frac{1}{{R_{S} }}} \right) + \frac{1}{b}log_{10} \left( {\frac{{ln\left( {u_{S} } \right)}}{ln\left( u \right)}} \right)$$

This produces a form for the differential maximum magnitude that is reminiscent of Båth’s law (Eq. 3), where 1*/R*_*S*_ = *N/N*_*S*_ = 1 + *N*_*T*_*/N*_*S*_ = 1 + *R*_*TS*_. Here we note that ΔM and $R_{S}$ are physically observable quantities that may be subjected to hypothesis testing.

In continuing to develop our model, we assume a further constraint on the stimulated and trailing earthquake counts *N*_*S*_ and *N*_*T*_. Similar to the prior logic in Båth’s law, we bring additional quantification to the value of $R_{S}$. First, the stimulated earthquake count *N*_*S*_ has been suggested to have a linear relationship with injection volume^32^.

6 $$N_{S} = V\left( t \right) 10^{\Sigma } 10^{ - bM}$$

This expression effectively modifies the *a*-value of the GR-MFD to have an explicit dependence on the volume injected *V*(*t*) (in m^3^) and the background potential for earthquakes i.e., the seismogenic index *Σ*. This relationship assumes a non-homogenous Poisson process for the stimulation events^33^ and has been well fit to numerous induced earthquake sequences^29,34,35^. Second, the total aftershock count is the integration of an aftershock rate decay function from the time of the mainshock to infinite time. In practice, induced sequences have often been observed to fit aftershock-like rate decay sequences following shut-in^36^. While there is evidence to suggest a linear dependence between injected volume and cumulative earthquake counts^29^, we note that alternative models^7,37^ could be utilized without changing the results of our study. Because there is an abundance in the choice of aftershock rate decay model, we summarize the prominent ones in Table 1. For each of the aftershock rate decay models we provide the rate decay function $n\left( t \right)$, the cumulative count function $N\left( t \right)$, and the total aftershock count $N\left( \infty \right)$. We also refer the reader to prior summaries of tectonic aftershock models^38^. In this study we included the most general version of aftershock models that (with parameter constraints) lead to finite cumulative event counts $N\left( \infty \right)$ and are widely adopted by the seismological community.

**Table 1:** Aftershock models proposed for tectonic earthquakes.

Model name [References]	Aftershock rate decay	Cumulative aftershock count	Total aftershock count
Modified Omori^39^	$n^{o} \left( t \right) = K\left( {t + c} \right)^{ - p}$	$N^{o} \left( t \right) = \left( {\frac{K}{p - 1}} \right)\left( {c^{1 - p} - \left( {t + c} \right)^{1 - p} } \right)$	$N^{o} \left( \infty \right) = K\frac{{c^{1 - p} }}{p - 1}$
Exponential^40^	$n^{e} \left( t \right) = n^{e} \left( 0 \right)e^{ - t/\tau }$	$N^{e} \left( t \right) = \tau n^{e} \left( 0 \right)\left( {1 - e^{ - t/\tau } } \right)$	$N^{e} \left( \infty \right) = \tau n^{e} \left( 0 \right)$
Stretched exponential^41^	$n^{s} \left( t \right) = q N^{\star} e^{{\left( {\frac{d}{{t_{0} }}} \right)^{q} }} \left( {\frac{1}{t + d}} \right)\left( {\frac{t + d}{{t_{0} }}} \right)^{q} e^{{ - \left( {\frac{t + d}{{t_{0} }}} \right)^{q} }}$	$N^{s} \left( t \right) = N^{\star} \left( {1 - e^{{\left( {\frac{d}{{t_{0} }}} \right)^{q} }} e^{{ - \left( {\frac{t + d}{{t_{0} }}} \right)^{q} }} } \right)$	$N^{s} \left( \infty \right) = N^{\star}$
Cut-off Power Law^42^	$n^{c} \left( t \right) = K\left( {t + c} \right)^{ - p} e^{ - t/\tau }$	$N^{c} \left( t \right) = K\tau^{1 - p} e^{c/\tau } \left( {{\Gamma }\left( {1 - p,\frac{c}{\tau }} \right) - {\Gamma }\left( {1 - p,\frac{t + c}{\tau }} \right)} \right)$	$N^{c} \left( \infty \right) = K\tau^{1 - p} e^{c/\tau } {{ \Gamma }}\left( {1 - p,\frac{c}{\tau }} \right)$
Gamma^43^	$n^{\gamma } \left( t \right) = At^{ - q} \left( {{\upgamma }\left( {q,\lambda_{b} t} \right) - {\upgamma }\left( {q,\lambda_{a} t} \right)} \right)$	$N^{\gamma } \left( t \right) = \left( {\frac{A}{q - 1}} \right)\left( {\left[ {\lambda_{b}^{q - 1} - \lambda_{a}^{q - 1} } \right] - \left[ { - \lambda_{a}^{q - 1} e^{{ - \lambda_{a} t}} + t^{1 - p} {{ \Gamma }}\left( {q,\lambda_{a} t} \right) + \lambda_{b}^{q - 1} e^{{ - \lambda_{b} t}} - t^{1 - p} {{ \Gamma }}\left( {q,\lambda_{b} t} \right)} \right]} \right)$	$N^{\gamma } \left( \infty \right) = \left( {\frac{A}{q - 1}} \right)\left( {\lambda_{b}^{q - 1} - \lambda_{a}^{q - 1} } \right)$
Epidemic type^44^	$n^{E} \left( t \right) = \sum\nolimits_{i} {K_{i} } \left( {t - t_{i} + c} \right)^{ - p}$	$N^{E} \left( t \right) = \sum\nolimits_{i} {\left( {\frac{{K_{i} }}{p - 1}} \right)} \left( {c^{1 - p} - \left( {t - t_{i} + c} \right)^{1 - p} } \right)$	$N^{E} \left( \infty \right) = \sum\nolimits_{i} {K_{i} } \frac{{c^{1 - p} }}{p - 1}$

For each model we provide an expression for the aftershock rate function $n\left( t \right)$, the cumulative aftershock count function $N\left( t \right)$, and the total number of aftershocks $N\left( \infty \right)$. Corresponding references are also provided for each model. Note that ${\upgamma }\left( {a,x} \right)$ and ${\Gamma }\left( {a,x} \right)$ are the lower and upper incomplete gamma functions, respectively. Superscripts are used to denote the aftershock decay model used.

We can expand upon Eq. (5) by imposing our archetypal induced earthquake sequence (Fig. 1c) in combination with the aftershock and stimulation models. Combining these models with the archetypal sequence allows for closed form expression of $R_{TS}$, and thus the distribution of the expected maximum earthquake magnitude.

7 $$R_{TS}^{o} = \frac{{N_{T} }}{{N_{S} }} = \frac{{\dot{V}\left( t \right) {\Delta T} 10^{\Sigma } + K\frac{{c^{1 - p} }}{p - 1}}}{{\dot{V}\left( t \right) T_{S} 10^{\Sigma } }} = \frac{{\dot{V}\left( t \right) {\Delta T} 10^{\Sigma } + f \dot{V}\left( t \right) 10^{\Sigma } \frac{c}{p - 1}}}{{\dot{V}\left( t \right) T_{S} 10^{\Sigma } }} = \frac{{ {\Delta T} + f\frac{c}{p - 1}}}{{ T_{S} }}$$

8 $$R_{TS}^{e} = \frac{{N_{T} }}{{N_{S} }} = \frac{{\dot{V}\left( t \right) {\Delta T} 10^{\Sigma } + \tau n^{e} \left( 0 \right)}}{{\dot{V}\left( t \right) T_{S} 10^{\Sigma } }} = \frac{{\dot{V}\left( t \right) {\Delta T} 10^{\Sigma } + f \dot{V}\left( t \right) 10^{\Sigma } \tau }}{{\dot{V}\left( t \right) T_{S} 10^{\Sigma } }} = \frac{{ {\Delta T} + f\tau }}{{ T_{S} }}$$

Here, $R_{TS}$ has been described for both the Modified Omori ($R_{TS}^{o}$, Eq. 7) and Exponential ($R_{TS}^{e}$, Eq. 8) aftershock rate decay models. Note that we’ve assumed the initial aftershock rate is some fraction *f* of the ending stimulation rate—to account for slight increases or decreases in earthquake rate after the lagged shut-in response.

## Testing the model’s validity

In adapting Båth’s law to induced seismicity, we’ve made several assumptions (Fig. 1): that the sequence is described by a nonhomogenous Poisson process, that the extended fault system can effectively be modelled as a point-process, that there’s a single lag time between operations and seismogenic response, that statistical parameters are stationary during the observation interval, and that there are no injection-volume or tectonic limits on the maximum magnitude. To validate (or refute) these assumptions, we begin by examining induced seismicity cases.

We focus only on cases where there has been a clearly delineated end of operations (Fig. S1). This criterion dominantly encompasses cases related to hydraulic fracturing^2^ or enhanced geothermal systems^45^. We select cases of induced seismicity where both catalogue and injection time series information is available. Cases include the Duvernay^11,13,46,47^ and Montney^48^ in Canada, the Bowland Shale in the UK^49,50^, the Appalachian Basin^51,52^, SCOOP & STACK play^53–55^, Eagle Ford^56^, and Arkoma Basin^57^ in the United States, the Sichuan Basin in China^6,58,59^, Soultz-sous-Forêts in France^60,61^, Cooper Basin^62^ and Paralana^63^ in Australia, KTB^64^ and Groß Schönebeck^65^ in Germany, Basel^66^ and St Gallen^67^ in Switzerland, Helsinki in Finland^68^, Pohang in South Korea^5,69^, and Berlín in El Salvador^70^. Many of these cases have data available in online repositories^71^, within aggregate comparison studies^12,29,72^, or as supplements to the individual case studies.

Briefly, individual cases are truncated at their magnitude of completeness (M_C_) and then subdivided into two populations: those occurring during stimulation or those trailing stimulation. We report statistics such as earthquake counts, largest magnitudes, *b*-value, and M_C_, which we summarize in Table S1. For additional details on the data acquisition and preprocessing, we direct the reader to the Supplementary Information (Section S1).

### Examining the Statistics of R_S_

**Figure 2:**
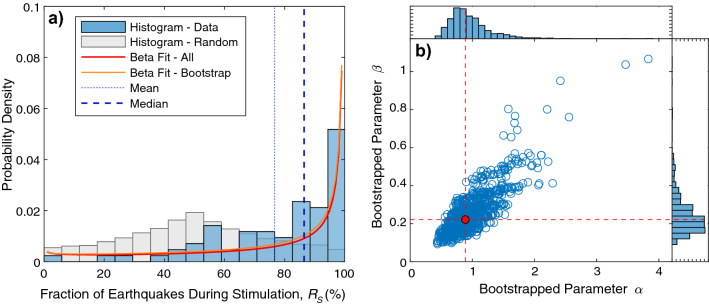
The empirical distribution of $R_{S}$ and bootstrapped beta distribution fit. (**a**) Histogram of $R_{S}$ for all the induced seismicity cases (blue bars) with its median and mean values (vertical lines), best fit to the beta distribution (crimson lines), compared against a random distribution (grey bars). (**b**) Scatterplot of bootstrapped beta distribution parameters (blue circles) with the best fit parameter (red circle). Inset histograms show the univariate distribution of beta parameters.

From our compiled database (Table S1), we examine the parameter $R_{S}$ observed during numerous induced seismicity sequences (Fig. 2a). $R_{S}$ shows a highly skewed distribution—tending towards values of 77% on average and 86% as a median (Fig. S5). This is comparable to observations in prior studies on hydraulic fracturing that found ~ 90% of events occurred during stage stimulation in the Duvernay^35^ and ~ 87% of events in the Sichuan Basin were ‘forced’ under a modified Epidemic Type Aftershock Model^6,58^. While the distribution is skewed towards high $R_{S}$ values, we also note that it is heavy-tailed with a handful of cases with low $R_{S}$ values. For example, the enhanced geothermal stimulation at Pohang shows a large trailing aftershock sequence^5,69^.

To further examine the skewed nature of the $R_{S}$ distribution, we compare our empirical observations to the distribution expected from a random process (Fig. 2a). To model the random process, we consider a uniform random variable for both *N*_*T*_ and *N*_*S*_. We then perform the two-sample Kolmogorov–Smirnov test between our empirically observed $R_{S}$ and the random process model. The two-sample Kolmogorov–Smirnov test is a non-parametric hypothesis test that assesses the probability that two samples are drawn from the same (arbitrary) distribution^73^. In performing this test, we find extremely small *p* values that are orders of magnitude less than 5% (Fig. S6)—which rejects the null hypothesis that the two samples are from the same distribution. The failure of $R_{S}$ to be modelled as a random process suggests that there is a physical process underlying this distribution (e.g., Eqs. 7 and 8).

A better representation of the statistics of the $R_{S}$ would be useful for estimation of differential magnitudes ΔM (e.g., Eq. 5). Towards this end, we fit our histogram results to a beta distribution—a two-parameter (α and β) family of distributions defined over the interval 0–1^74^. We select the beta distribution due to its flexibility in fitting probability density functions over this interval. Further, to capture the variability in the fitted parameters we performed a 1000-trial weighted bootstrap analysis (see Supplementary Information Section S3 for more details). We find that the mean best-fit parameters for α and β are 1.016 and 0.271, respectively. Similarly, the median best-fit parameters for α and β are 0.940 and 0.238, respectively. The covariances (σ_αα_, σ_ββ_, σ_αβ_) for these parameter fits are 0.1151, 0.0130, and 0.0317 (Fig. 2b). In performing sensitivity tests, we do not find that fitted parameters differ appreciably, when comparing hydraulic fracturing or enhanced geothermal system subsets. However, larger datasets could be useful to discern systematic differences in $R_{S}$ according to operation and setting. These fitted $R_{S}$ distribution results could constrain trailing seismicity differential magnitudes ΔM, provided the validity of using $R_{S}$ in Båth’s law for induced seismicity was established.

### Testing Båth’s law for induced seismicity

With constraints on the empirical distribution of $R_{S}$, we examine the statistical validity of Båth’s law for induced seismicity. Equation (5) predicts that there is a linear relationship between the trailing-stimulation maximum magnitude difference ΔM (left hand side of equation) and the *b*-value scaled logarithm of the population ratio $\frac{1}{b}log_{10} \left( {R_{TS} } \right)$ (first term of the right-hand side), where the remaining term describes the statistical distribution of the residuals (second term of right-hand side).

**Figure 3:**
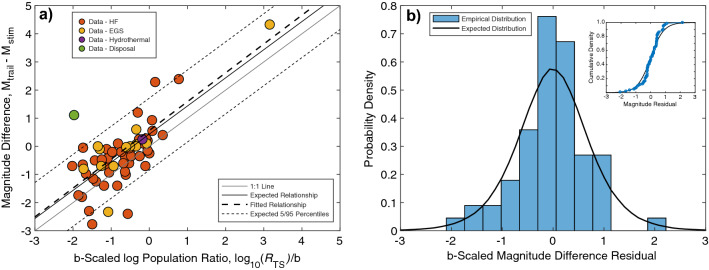
Testing Båth’s law for induced seismicity. (**a**) Linear regression of magnitude differences ΔM versus logarithmic population ratios $\frac{1}{b}log_{10} \left( {R_{TS} } \right)$: data is separated by anthropogenic type (circles) and shown alongside the 1:1 line (solid gray line), the relationship expected by Båth’s law (solid black line), the regression fit to data (dashed line), and the corresponding 5/95 percentiles (dotted line). (**b**) The residuals to the expected Båth’s law relationship are shown as probability density function for the expected (solid black line) and empirically derived from data (blue bars). Inset panel shows the cumulative density function for the expected (solid black line) and empirically derived residuals (blue circles/line).

Based on this, we first consider a weighted linear regression applied to our dataset (Fig. 3a). We observe a reasonable agreement between observation and expectation. The fitted parameters for the linear regression are 1.06 ± 0.16 for the slope and 0.58 ± 0.18 for the y-intercept. The theoretically expected slope of 1.0 agrees within error of our regression-fitted slope (see Supplementary Information Section S4 for more details). The observation of a y-intercept term is the result of a correction term that conditions on the sample count, when there is an unknown true sample count (see van der Elst et al.^29^ Sections 2.2 & A4 for additional details). The value of this correction term approaches $\frac{1}{b}log_{10} \left( e \right)$ (i.e., ~ 0.43) as the population count *N* becomes large, which agrees within error of our regression fitted parameter. While both the empirically derived slope and y-intercept agree within error to their expected values, we note that the best estimate of both values are higher than those expected; this larger value bias could be the result of a publication bias where scientific studies preferentially describe cases with ‘exceptional behavior’, like large magnitudes, red-light events, or lingering sequences of trailing seismicity.

The second test we consider is an examination of the distribution of the Båth’s law residuals (Fig. 3b). In this test, we rearrange the terms in Eq. (5) such that just the power law distribution remains: i.e., $log_{10} \left( {\frac{{ln\left( {u_{S} } \right)}}{{ln\left( {u_{T} } \right)}}} \right)$. Note that individual correction terms are accounted for using the true population size, rather than the large *N* limit. We find a correspondence between the histogram of data residuals and the expected probability density function for Båth’s law. More quantitatively, we compare the expected and observed residual samples using the two-sample Kolmogorov–Smirnov test. We find a *p* value (~ 45%) that is larger than 5%, meaning we are unable to reject the null hypothesis that the two samples are from the same distribution. Most bootstrapped perturbations (~ 61%) support this result (Fig. S7). Of course, additional high-resolution cases of induced seismicity would be helpful for further scrutiny. The observation that induced earthquakes trailing the stimulation follow Båth’s law, both for expected magnitudes versus population counts, and for their residual distribution suggests that this approach should be useful for evaluating trailing seismicity.

## Discussion

In this section we discuss the interpretations and implications of our results. We divide the discussion into three parts: (1) the applicability of Båth’s law for induced seismicity, (2) the relative importance of parameters in Båth’s law, and (3) a diagnostic test for operators to use in situ when encountering seismogenic faults.

### The applicability of Båth’s law for induced seismicity

To date, many models have been introduced to capture the anticipated maximum magnitude of induced earthquakes from either physical or statistical perspectives^7,29,37^. Typically, these models have focused on the earthquakes occurring during the stimulation process. In this paper, we have described a statistical framework to extend these considerations to those earthquakes that trail after well shut-in based on Båth’s law for tectonic earthquakes. This conceptual extension is partly driven by the observation of large earthquake magnitude jumps^13^, high-profile cases of large trailing events^5,11,12,48^, and the contribution of trailing events to earthquake risk management^9^.

Our results suggest that Båth’s law is a viable foundation for anticipating the magnitude of earthquakes trailing an operation that produces anthropogenic seismicity. This assertion is supported by the observation that: (1) induced seismicity cases are well fit by the linear relationship between magnitude differences ΔM and logarithmic population ratios $\frac{1}{b}log_{10} \left( {R_{TS} } \right)$ that is predicted by Båth’s law (Fig. 3a), and (2) the residuals from this relationship are approximately consistent with the power law distribution anticipated by Båth’s law (Fig. 3b). Based on this, we further suggest that the description of Båth’s law (Eq. 5) will have predictive power for quantitative forecasts of trailing seismicity. Of course, this is predicated on knowing/estimating the population ratios $R_{TS}$—either a priori or updating in real-time during an operation. In this sense, the empirical distribution of $R_{S}$ and its best fit to a beta distribution (Fig. 2) provides some utility—possibly as prior information in a Bayesian updating scheme. Much of the literature that has been described for tectonic aftershock forecasts through extreme value statistics and Bayesian approaches^22,75^, will likely be adaptable for the purpose of constraining *N*_*T*_. Additionally, models of stimulation seismicity that consider the full injection history should provide better predictability to the anticipated stimulation events *N*_*S*_^50^.

This interpretation is in line with recent work that has found that induced earthquake sequences have statistical properties that are similar to (or subtle modifications of) their tectonic counterparts^29,36^. These results could be indicative of the importance of fluid-flow along faults for tectonic earthquakes, which has been suggested in some studies as important factor for the aftershock process^76,77^. General links between statistical models and physical processes have been made for earthquakes and aftershocks^78^. Thus, incorporating and testing physical models of aftershocks that consider realistic fault geometries, fault rheology, stress, material heterogeneity, and fluid flow will likely be important for better understanding post shut-in induced earthquakes.

### Relative importance of Båth’s law parameters

A better understanding the physical processes that underlie the observed statistics would allow for better understanding of trailing seismicity. While the collected dataset (Table S1) is too small to meaningfully discern systematic trends in $R_{S}$ that vary with anthropogenic type, operational parameters, fault architecture, or geological setting, we may still apply our simple model to better understand the relative importance of parameters that contribute to a skewed distribution of $R_{S}$. We focus first on the exponential decay model (Eq. 8) both for its simplicity and the breadth of induced seismicity studies it has been applied to^79,80^. If we assume representative values for a hydraulic fracturing operation (ΔT = 1 h, $\tau$ = 3 days, $f$ = 1.0, *T*_*S*_ = 2 weeks), we find that $R_{S}$ = 82%; a value between those reported for the mean/median of the empirical $R_{S}$. To build on this further, we assume that the parameters ΔT and $\tau$ follow a lognormal distribution while $f$ follows a Gaussian distribution so that we may compute a synthetic $R_{S}$ distribution. From this, we perform a Markov Chain Monte Carlo (MCMC) optimization to maximize the Kolomogorov–Smirnov *p* value between the empirical and synthetic $R_{S}$. The MCMC approach randomly samples the parameter space to find optimal mean and standard deviations of these parameters (ΔT, $\tau$, $f$) that are bounded within those reasonably observed in the literature^2,79^.

**Figure 4:**
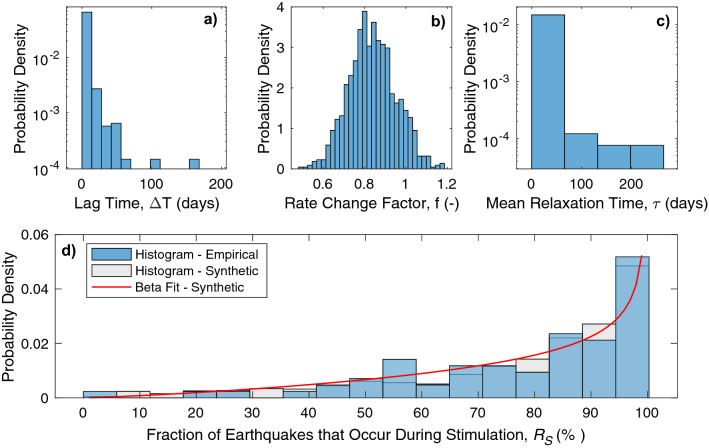
Synthetically fitting the $R_{S}$ distribution to model parameters. (**d**) The archetypal model for trailing seismicity ratios (Eq. 8) is fit (grey bars) to the empirical distribution of $R_{S}$ (blue bars). The distribution of model parameters (**a**) ΔT, (**b**) *f*, and (**c**) $\tau$ are also shown.

The MCMC approach produces synthetic $R_{S}$ distributions that fit the empirical distribution well (likelihoods of up to 90%). The input parameters distributions needed to fit $R_{S}$ well (Fig. 4) suggests that a skewed distribution of input parameters (ΔT or $\tau$) is required to match the skewed $R_{S}$ distribution. Such a skewed distribution includes chances for black swan occurrences of extreme value events. In practice, this is what is observed in cases of induced seismicity: relatively rare events of large consequence. For example, the earthquakes at Pohang were exceptional in that a dormant fault system was reactivated by the geothermal stimulation program^5^. Similarly, most events in a 2018 episode in British Columbia occurred as part of a delayed trigger after hydraulic fracturing stage completion^48^. The largest event during the second well stimulation at Preston New Road occurred 60 h after the end of the last stimulation^50^. Earthquakes were apparently induced at distances of up to ~ 5 km away from the enhanced geothermal stimulation at Strasbourg-Vendenheim^81^. These consequential trailing seismicity events suggest the importance of better modelling and constraining parameters relevant to Båth’s law. We note, however, that there are significant limitations to our MCMC approach, as the distributions of ΔT and $\tau$ trade-off with one another. Thus, empirical observations of aftershock parameters ($\tau$ or *c* and *p*) and injection responses (*Σ*, *b*, ΔT) would be needed to better constrain these values/distributions.

### The Seismogenic fault injection test (SFIT)

Previously we showed that skewed values of ΔT or $\tau$ are required for a skewed $R_{S}$—and represent an increased potential for large trailing magnitude differences ΔM. Given the possibility of consequential impacts from large magnitude trailing events, it would be prudent to measure these parameters in situ to reduce the uncertainties associated with fault reactivation.

**Figure 5:**
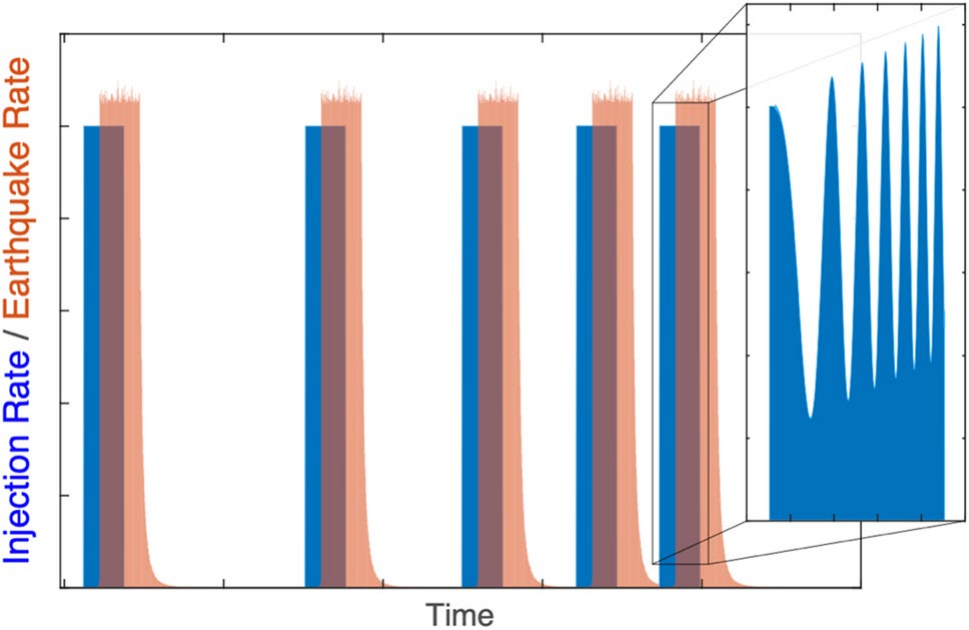
The Seismogenic Fault Injection Test (SFIT). Schematic diagram of a diagnostic test to measure a fault’s seismogneic response through parameters such as *Σ*, *b*, ΔT, and $\tau$ (or *c* and *p*). Repeated injection tests (at non-periodic intervals) are performed to verify the accuracy of measurements. Inset diagram shows how individual tests could be ‘chirped’ in their injection rate to better constrain parameters.

Similar to Diagnostic Fracture Injection Tests^82^ where fluids are intentionally injected into a reservoir to constrain its hydrological properties and stress state, we suggest an analogous Seismogenic Fault Injection Test (SFIT) to calibrate the seismic response to fluid injection (Fig. 5). Ideally, an inventory of faults would be available from a pre-assessment to injection. Injection would be performed with the intention of measuring both the time-delayed response to injection (*Σ*, *b*, ΔT) and the aftershock-like decay of seismicity ($\tau$ or *c* and *p*) for each of the known faults^79^. The SFIT should be designed to constrain parameters of greatest consequence (ΔT and $\tau$). In the context of our study, unknown faults activated at distance from the stimulation interval (e.g., large ΔT) or with prolonged trailing sequences (e.g., large $\tau$) pose the greatest risks. With this in mind, an individual injection phase would span a range of injection rates, potentially sweeping through them cyclically in a ‘chirped’ manner (Fig. 5); the intent would be to both discern any rate-dependent features and better highlight potential time lags ΔT. As well, individual injection phases should be repeated to bolster confidence in the measurements, in particular the decay rate $\tau$ which only is well constrained after shut-in^79^. Ideally, these repeated injections phases would be spaced at non-periodic intervals to unambiguously measure delay times ΔT^83,84^. Finally, if we are making suggestions outside of what the simple models predict, varying the shut-in procedure for individual injection phases would be useful to discern safer responses to unwanted seismicity.

## Conclusions

In summary, we have tested the applicability of Båth’s law to cases of induced seismicity with clear ends to fluid injection. Båth’s law fits the expected relationship, as confirmed by a linear regression of the collected data. Additionally, the residuals to the data follow the expected power law distribution, as confirmed by Kolomogorov–Smirnov tests. Thus, our compilation of population ratio $R_{S}$ statistics, including a fit to the beta distribution, could be useful for forecasting trailing seismicity. Finally, simple point-like models describing $R_{S}$ highlighted parameters of interest (ΔT and $\tau$); we propose in situ tests to help quantify the risks associated with seismicity trailing an anthropogenic operation. Variables used throughout the paper are summarized in Table 2.

**Table 2:** Summary of equation parameters and variables.

Variable	Description
*a*	GR-MFD productivity parameter, *a*-value
*b*	GR-MFD slope parameter, *b*-value
${\text{M}}$, ${\Delta M}$	Earthquake magnitude and magnitude difference, respectively
${\text{M}}_{{\text{C}}}$	Magnitude of completeness
*Σ*	Seismogenic Index
$V\left( t \right)$, $\dot{V}\left( t \right)$	Cumulative injection volume and injection rate, respectively
$n\left( t \right)$	Earthquake rate
$N$, $N_{A}$,$N_{T} , N_{S}$	Cumulative earthquake counts: total, aftershocks, trailing, and stimulation
$R_{S}$, $R_{TS}$	Earthquake count ratios: related by 1/*R*_*S*_ = *N*/*N*_*S*_ = 1 + *N*_*T*_/*N*_*S*_ = 1 + *R*_*TS*_
$u$, $u_{T}$,$u_{S}$	Confidence level variables
${\Delta T}$	Simple archetype model, stimulation-earthquake response delay
$T_{S}$	Stimulation/injection time interval
$f$	Stimulation-trailing rate change factor
*K*	Omori aftershock productivity parameter
*p*	Omori aftershock decay exponent
*c*	Omori aftershock singularity parameter
$\tau$	Exponential aftershock mean decay time

Here we list the relevant parameters used in equations throughout our study, for convenience to the reader.

## Supplementary Information

Supplementary Information 1.

Supplementary Information 2.

## Data Availability

Data used to produce the results of this study are available as an electronic supplement (Table S1). In addition, the codes used to derive these results are available online, through GitHub (https://github.com/RyanJamesSchultz/IS-Bath). Catalogue data and injection data are available in online repositories ^71^, within aggregate comparison studies^12,29,72^, or as supplements to the individual case studies.
