# Supplementary material for: Statistical bounds on how induced seismicity stops
Source: Sci Rep. 2022 Jan 24;12:1184. doi: 10.1038/s41598-022-05216-9 (PMC8786864; doi:10.1038/s41598-022-05216-9)
Supplement: Supplementary file 1 — Supplementary Information 1. [file 41598_2022_5216_MOESM1_ESM.docx]

**Supplementary Information for the article**

“Statistical bounds on how induced seismicity stops”

*by*

*Ryan Schultz, William L. Ellsworth, & Gregory C. Beroza*

**published in**

**
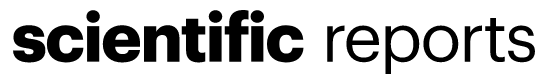
**

**Contents of this file**

Supplementary Text S1 to S5

Figures S1 to S8

**Additional Supporting Information (Files uploaded separately)**

Caption for Table S1

**Supplementary Text**

S1. Catalogue/Injection Data Preprocessing

To populate the contents of Table S1, we begin by identifying ~80 cases of induced seismicity worldwide (Figure S1). From these cases, we ascertain information on the available catalogued events and injection rate time series data. We grade the datasets based on the quality/completeness of the information available: A for those with full catalogue and injection rate timeseries data, B for those with full catalogue information but only on/off times for injection, and C for those cases where information had to be gleaned either from the text or figures of a publication. We quality control the catalogue and injection data we compiled against each of the individual case studies, to ensure that the information is consistent with previously reported results.

Catalogue information is used to derive estimates of the Gutenberg Richter magnitude frequency distribution (GR-MFD) statistics including the *b*-value and magnitude of completeness (Mc). Magnitude of completeness is estimated as the cut-off value the maximizes the goodness-of-fit to the GR-MFD, within a band of magnitudes near the mode of the non-cumulative GR-MFD. Next, the *b*-value is estimated from this truncated set of earthquakes above the Mc using a maximum likelihood approach. We compare our results to those from the individual cases studies where the catalogue data was originally derived and find that our results are consistent with those of the parent studies.

To collect information relevant to Båth’s law, we subdivide the catalogue data into two partitions: those that occur during stimulation or those that occur outside of stimulation. In cases where Grade A data is available, this is simply defined as times when (positive) non-zero injection rates are occurring (Figure S2). In cases where Grade B data is available, this is simply defined as the “on” periods of injection (Figure S3). In cases where only Grade C data is available, we glean what information we can from text and figures (e.g., Figure S4); for example, by examining the relative proportionality of events occurring during a stimulation period. Note that we consider any type of injection as relevant to the seismogenic process – this includes operations like acid stimulation, drilling mud loss, or regular injection/stimulation. We do not consider the potential for lagged seismicity response: either following the start of injection or following the shut-in of operations. Finally, we consider the operation and seismicity as a point process, so no knowledge of the spatial extent of faulting or fault architecture is included in our partitioning.

These two partitions of data are examined to collect the information in Table S1. We simply observe the number of earthquakes above Mc (Nstim & Ntrail) and the largest magnitude events (Mstim & Mtrail). This process is repeated for both the stimulation/injection partition and the trailing ‘aftershock’ partition. Again, we quality control our results against the text and figures from the parent studies to ensure the veracity of our results. We also cross-checked our results against other induced seismicity comparison studies [*12,29,72*] to ensure consistency, as appropriate.

S2. Data Weighting Scheme

In anticipation of performing our statistical tests, we devise a weighting scheme to give a relative ranking of importance among the case studies. The dominant parameter considered between case studies is the catalogue population size *N*=Ntrail+Nstim. Here we use a weighting scheme as the fourth root of the population size *N^1/4^*. We justify this choice as a mild weighting scheme that compromises between no weighting (*N^0^*), and one based on the standard deviation for a Poissionian process (*N^1/2^*). Finally, we apply a multiplicative constant to the population-based weights based on the assigned grades: 1.1 for A, 1.0 for B, and 0.9 for C.

These weightings were utilized to place relative importance on case studies during statistical tests, parameter estimation, and bootstrapping. For example, our bootstrap approach is modified to use these weights by modifying the likelihood of retaining an event to be proportional to its weight during decimation – this way lower weighted cases are more likely to be removed and higher weighted cases are more likely to be retained. This weighted bootstrap approach will be utilized for multiple statistical tests.

S3. Testing and Bootstrapping of $R_{S}$

In Section 4.1 “Examining the Statistics of $R_{S}$”, we performed statistical tests to discern the nature of $R_{S}$ observed in induced seismicity cases. Here we elaborate on the details of these tests.

First, the skewed nature of $R_{S}$ was shown through the examination of mean and median values. To assess the robustness of this observation, we applied a 1000-trial weighted bootstrap procedure that retains 90% of the cases during each trial. Generally, the bootstrapped distributions follow the values reported from the full dataset (Figure S5). This same bootstrap procedure was also applied to the Kolmogorov-Smirnov test p-values when comparing the random and observed $R_{S}$ values. In all the bootstrap trials considered, the null hypothesis (that the observed and random $R_{S}$ samples are the same) is rejected (Figure S6).

Finally, our bootstrapping procedure was applied to the beta distribution fitting procedure of $R_{S}$ (Figure 2b). The beta distribution fitting process uses Matlab’s *betafit()* function and is repeated in the bootstrap trials. This process is first performed for the whole dataset, as reported in the main text in the manuscript. Additionally, we repeat this process for just those cases labelled as “hydraulic fracturing (HF)” and “enhanced geothermal systems (EGS)”. Fits to the parameters for the subsets of cases agree within reported errors.

S4. Testing and Bootstrapping Båth’s Law

In Section 4.2 “Testing Båth’s Law for Induced Seismicity”, we performed statistical tests to test the applicability of Båth’s law to induced seismicity cases. Here we elaborate on the details of these tests.

The first test considered was a linear regression between the trailing-stimulation magnitude difference $\Delta M$ and the *b*-value scaled logarithm of the population ratio $\frac{1}{b}{log}_{10}\left( R_{TS} \right)$. To perform this regression, we use a weighted least squares fit to the data. Weights are provided by our previously described scheme. Our previously described 1000-trail bootstrapping routine allows for estimation of the distribution of the parameter fit errors. Best fit parameters are considered by the fit to the full data. Standard error parameters are considered by examining the covariance of the fitted parameters. Overall, these fitted parameters agree within error of the expected values (see main text for more details).

The next test we considered was the two-sample Kolmogorov-Smirnov test applied to the residuals of Båth’s law. Similarly, we applied the 1000-trial weighted bootstrapping approach to the Kolmogorov-Smirnov test between the observed residuals and expected residuals (Figure 3b). We then examined the distribution of p-values that result from this test (Figure S7). The majority of cases are larger than the 5% statistical significance threshold, similar to the results for the full dataset. This suggests that there is no discernible difference between our observed residuals and those expected from Båth’s law.

Additionally, we tested the results of our analysis with the omission of the Pohang case, which is an outlier from the perspective of $R_{TS}$ (Figure 3a, top-right datapoint). We find that the same overall trends are noted: well-fitting linear regression parameters and residual distribution compared to the theoretical expectation.

S5. The Markov Chain Monte Carlo Approach

In Section 5.2 “5.2 Relative Importance of Båth’s Law Parameters”, we performed a random walk approach to optimize the distribution parameters for a synthetic $R_{S}$ distribution. Here we elaborate on the details of this approach.

Markov Chain Monte Carlo (MCMC) approaches sample a probability distribution. In our work, we are interested in deriving the empirical $R_{S}$ distribution from relationships expected from the exponential aftershock decay (Equation 8). We assume that input parameters $\Delta T$ and $\tau$ follow a lognormal distribution while $f$ follows a Gaussian distribution (and *T_S_* is fixed as a constant at 2 weeks). Thus, each of the parameters have a mean and standard deviation characterizing their distribution. From the parameter distributions, a synthetic $R_{S}$ distribution may be estimated according to our simple archetypal model (Equation 8). We bound the sampled parameter means to be between the given ranges: 0.0 days < $\Delta T$ < 1.0 days, 0.0 < $f$ < 1.5, 0.0 days < $\tau$ < 15.0 days. Also, we bound the sampled parameter standard deviations to be between the given ranges: 0.0 days < $\Delta T$ < 5.0 days, 0.00 < $f$ < 0.25, 0.0 days < $\tau$ < 5.0 days. The MCMC algorithm then randomly walks within these parameter bounds. For each iteration in this process we compute a synthetic $R_{S}$ distribution based on the current set of parameters. The synthetic and empirical $R_{S}$ are compared via the Kolomogorov-Smirnov test’s p-value, which we use as a likelihood metric in the MCMC approach. The MCMC approach then optimizes the result based on the Kolomogorov-Smirnov test’s p-value by keeping new samples with higher p-values and having to pass a randomized check for new samples with lower p-values. In this way, we sample the parameter space to find synthetic $R_{S}$ that are indistinguishable from the empirical ones.

Performing this MCMC test for 10^5^ iterations generally produces synthetic distributions with p-values of up to 90%. The best fitting set of parameters are chosen from the MCMC sampled path via a weighted average that’s weighted on the squared p-value (to better emphasize good fits to the data). These best fitting set of weighted average parameters are used for display and discussion in the main body text (Figure 4).

**Supplementary Table Caption**

**Table S1. Compiled induced seismicity data.** Data compiled for our study from several sources, with the data in each column explained below. Column A “ID” is a shorthand name for the induced earthquake sequence, column B “CID” is a numerical shorthand for the sequence, column C “Type” is the type of stimulation operation, column D “Country” is the country the earthquake occurred in, column E “Basin” is the basin the earthquakes occurred in, column F “Formation” is the formation targeted by the operator, column G “Nstim” is the number of earthquakes that occurred during stimulation, column H “Ntrail” is the number of trailing earthquakes that occurred outside of stimulation periods, column I “Fraction” is the ratio of stimulation events to the total count $R_{S}$, column J “Mstim” is the largest earthquake magnitude to occur during stimulation, column K “Mtrail” is the largest earthquake magnitude that occurred outside of stimulation periods, column L “b-value” is the GR-MFD *b*-value estimated from the data, column M “Mc” is the estimated (and used) magnitude of completeness, column N “Grade” is the quality of the derived dataset (A – full catalogue + injection data; B – full catalogue + on/off injection periods; C – derived from paper text/figures), column O “Reference” is the corresponding reference to the induced seismicity case study. The label “NaN” is used to indicate cases where portions of data were unavailable or unreportable.

**Supplementary Figures**

**
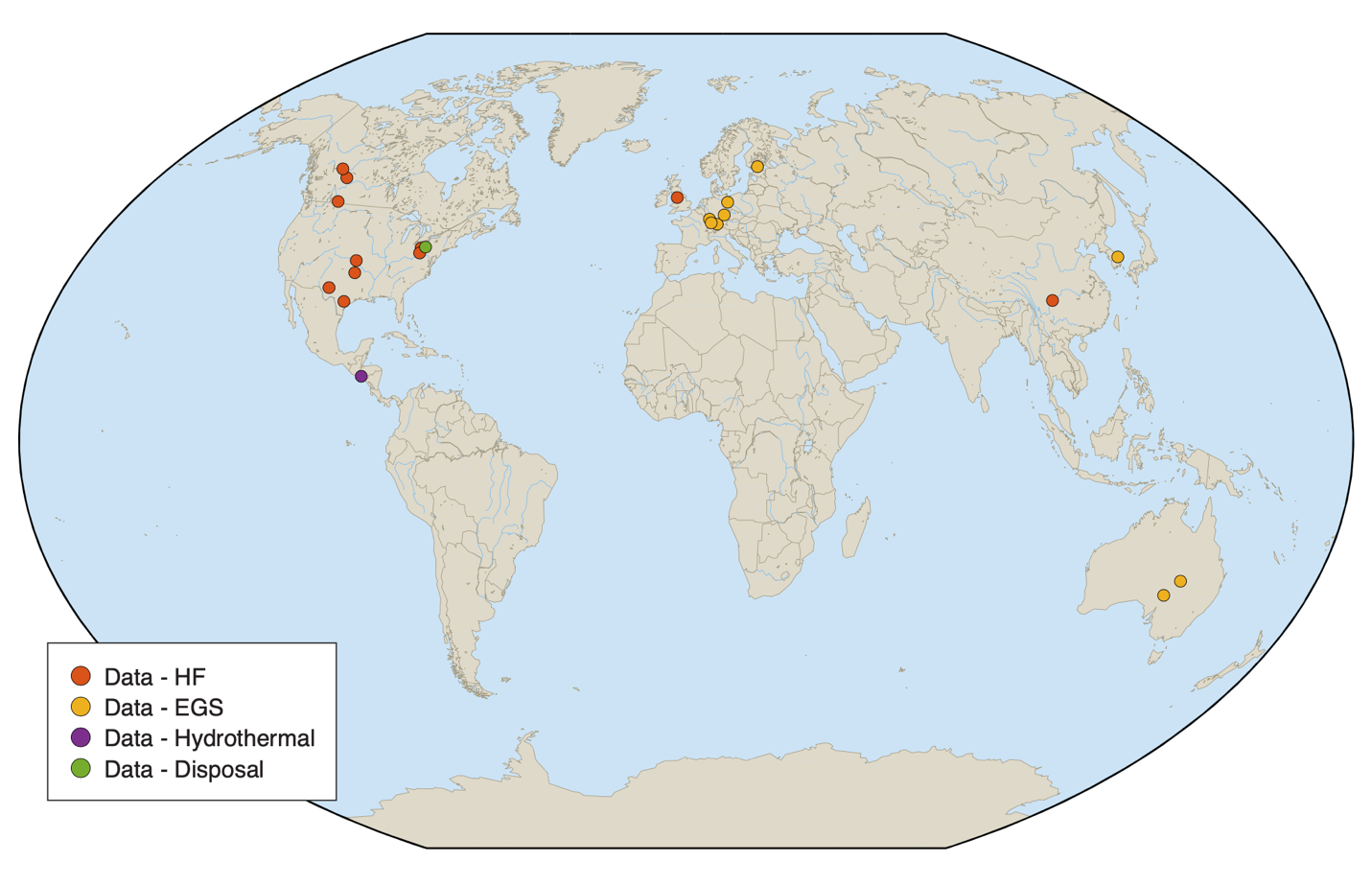
**

**Figure S1. Cases of induced seismicity worldwide.** Cases of induced earthquakes that are used in our study are shown with political boundaries for geographic context. Cases are coloured according to the anthropogenic operation that caused the earthquake.

**
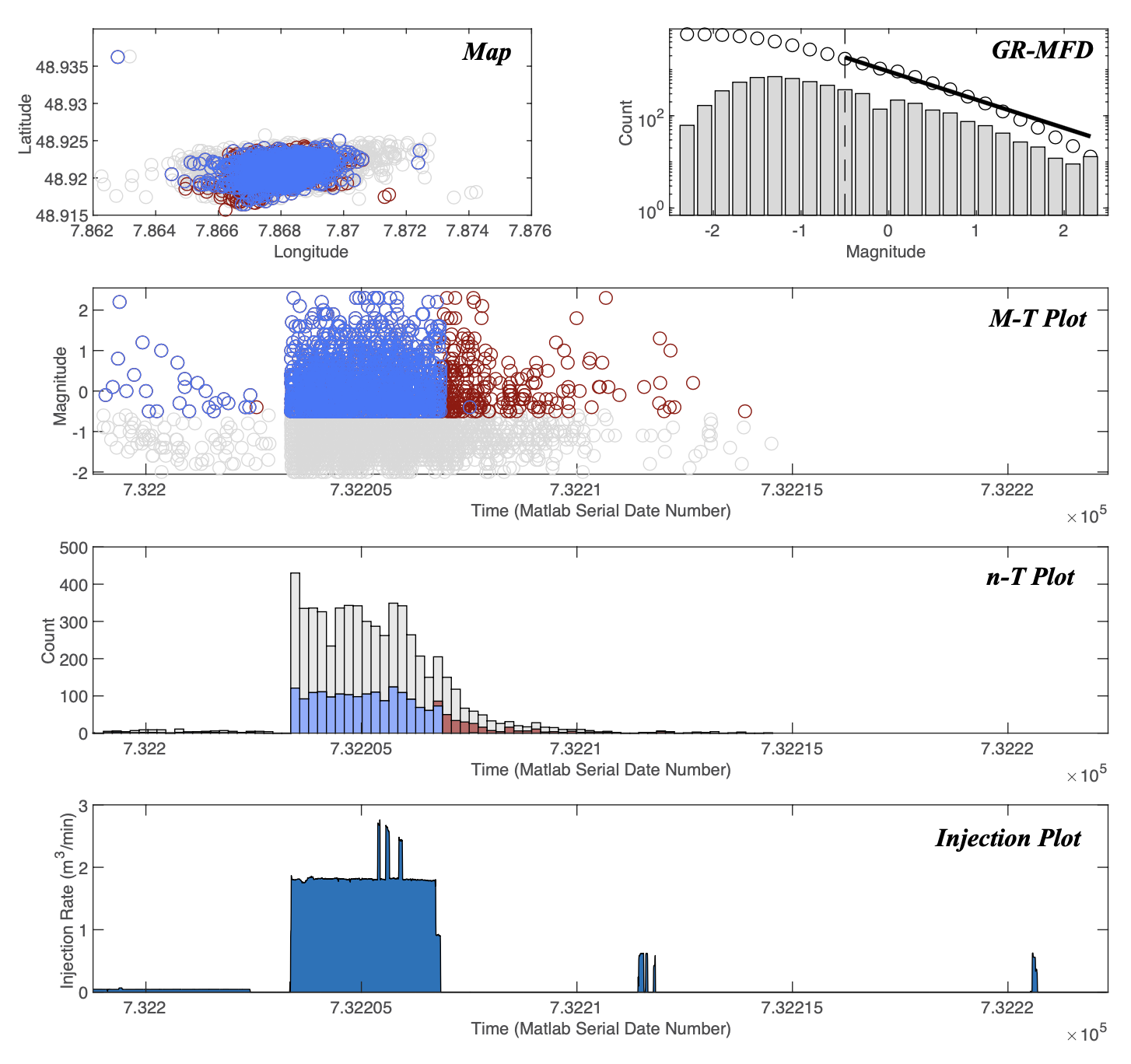
**

**Figure S2. Sample of the catalogue/injection preprocessing step (Grade A).** Data for the Soultz-sous-Forêts enhanced geothermal stimulation in 2004. Multiple panels convey information including a map of the earthquake locations (Map), the Gutenberg Richter frequency magnitude distribution (GR-MFD), earthquake magnitude as a function of time (M-T Plot), earthquake counts as a function of time (n-T Plot), and injection rate as a function of time (Injection Plot). In all panels, earthquakes partitioned into the injection subset are blue, trailing events are red, and those below the magnitude of completeness (Mc) are grey.

**
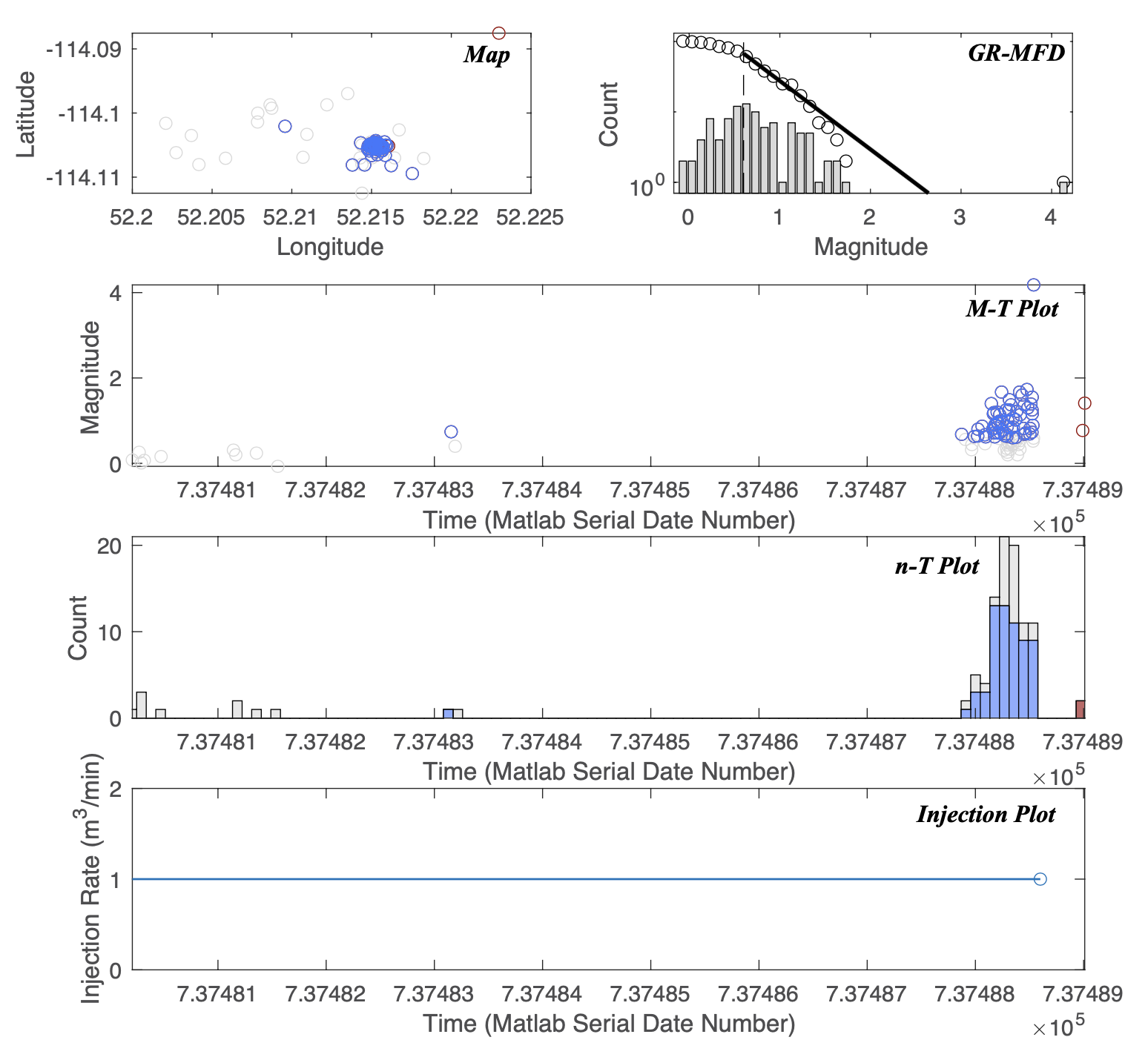
**

**Figure S3. Sample of the catalogue/injection preprocessing step (Grade B).** Data for the Duvernay East Shale Basin hydraulic fracturing in 2018. Multiple panels convey information including a map of the earthquake locations (Map), the Gutenberg Richter frequency magnitude distribution (GR-MFD), earthquake magnitude as a function of time (M-T Plot), earthquake counts as a function of time (n-T Plot), and injection on/off times (Injection Plot). In all panels, earthquakes partitioned into the injection subset are blue, trailing events are red, and those below the magnitude of completeness (Mc) are grey.

**
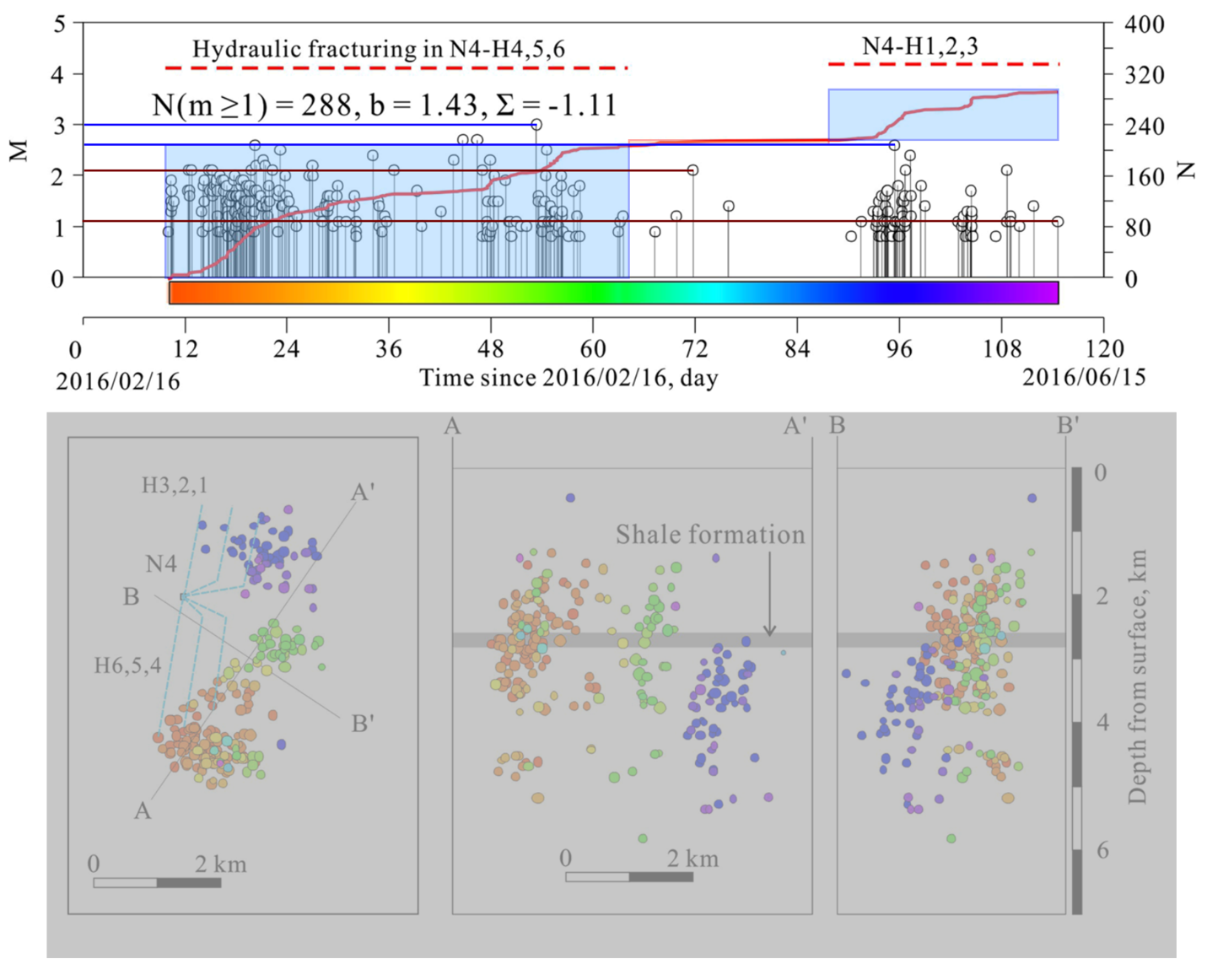
**

**Figure S4. Sample of the catalogue/injection preprocessing step (Grade C).** Example of data gleaned from figures in studies. The top panel shows a magnitude and cumulative count time plot with on/off injection times for hydraulic fracturing superimposed. Counts are derived by comparing the proportional heights of the added red/blue boxes. Magnitudes are derived by examining the projection of the added red/blue lines, from the largest earthquakes, onto the y-axis. Bottom panels are irrelevant for the analysis of our study. Base figure is reproduced from Lei et al., 2017 [*58*] figure 4.

**
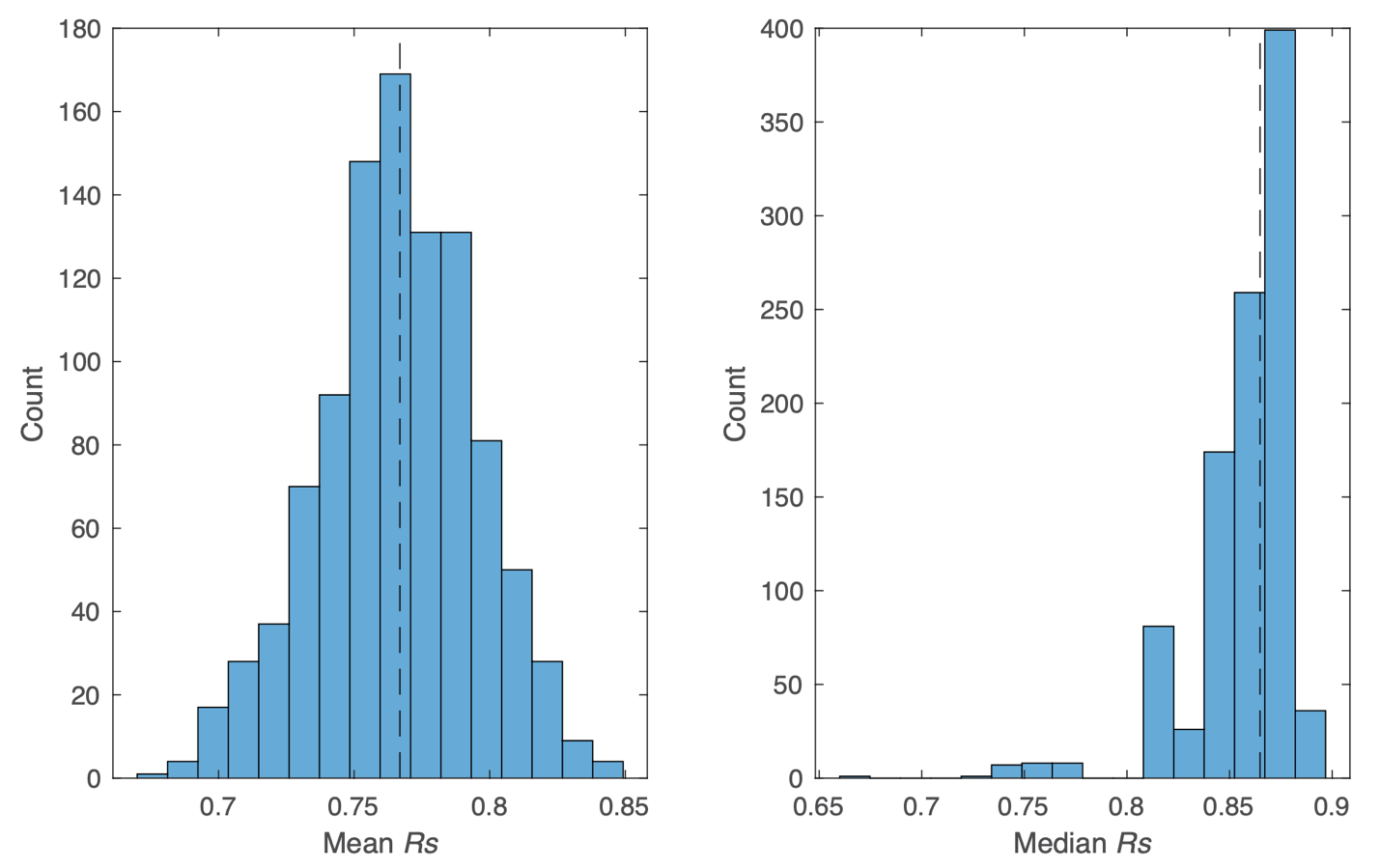
**

**Figure S5. Bootstrapped mean and median** $\boldsymbol{R}_{\boldsymbol{S}}$ **statistics.** Bootstrapped trials of the mean (left) and median (right) $R_{S}$ values are shown as a histogram (blue bars) on a log scale.

**
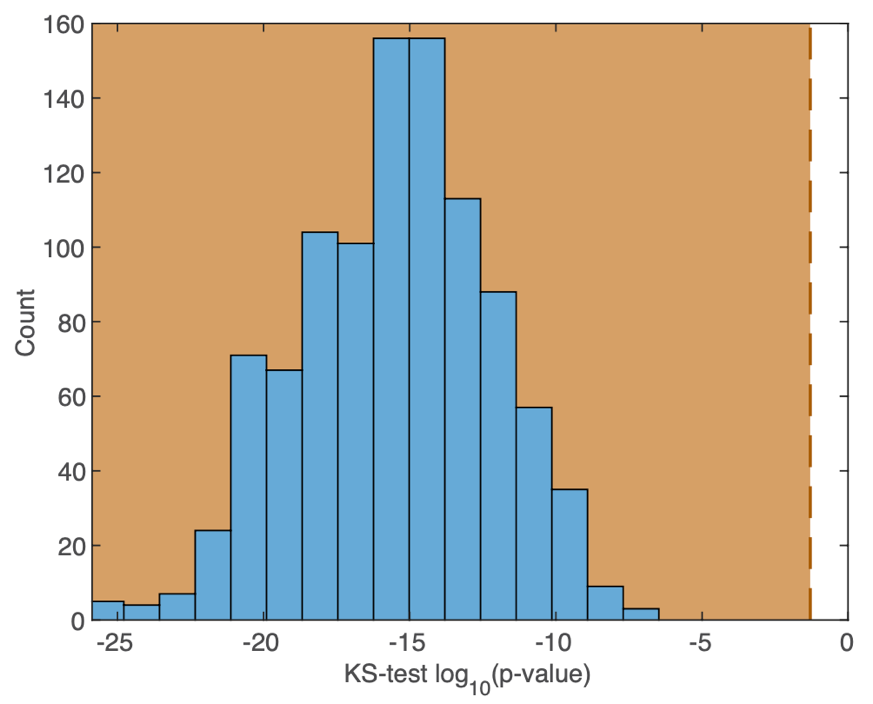
**

**Figure S6. Bootstrapped Kolmogorov-Smirnov test p-values for the** $\boldsymbol{R}_{\boldsymbol{S}}$ **statistic.** Bootstrapped trials of the p-value are shown as a histogram (blue bars) on a log scale. Orange coloured area indicates the region of 5% statistical significance.

**
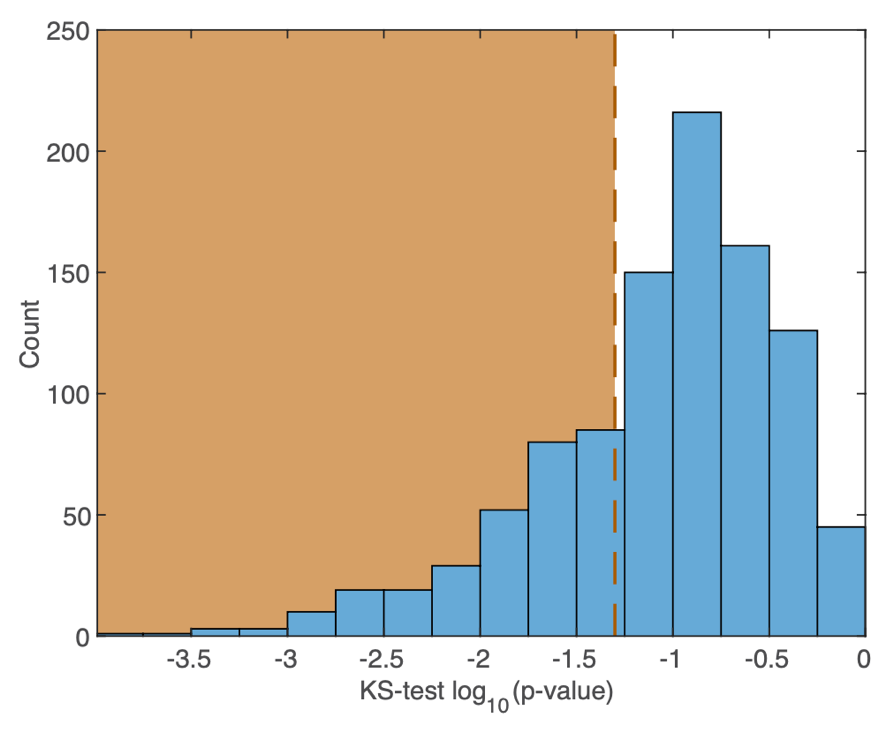
**

**Figure S7. Bootstrapped Kolmogorov-Smirnov test p-values for the Båth’s law residuals.** Bootstrapped trials of the p-value are shown as a histogram (blue bars) on a log scale. Orange coloured area indicates the region of 5% statistical significance.


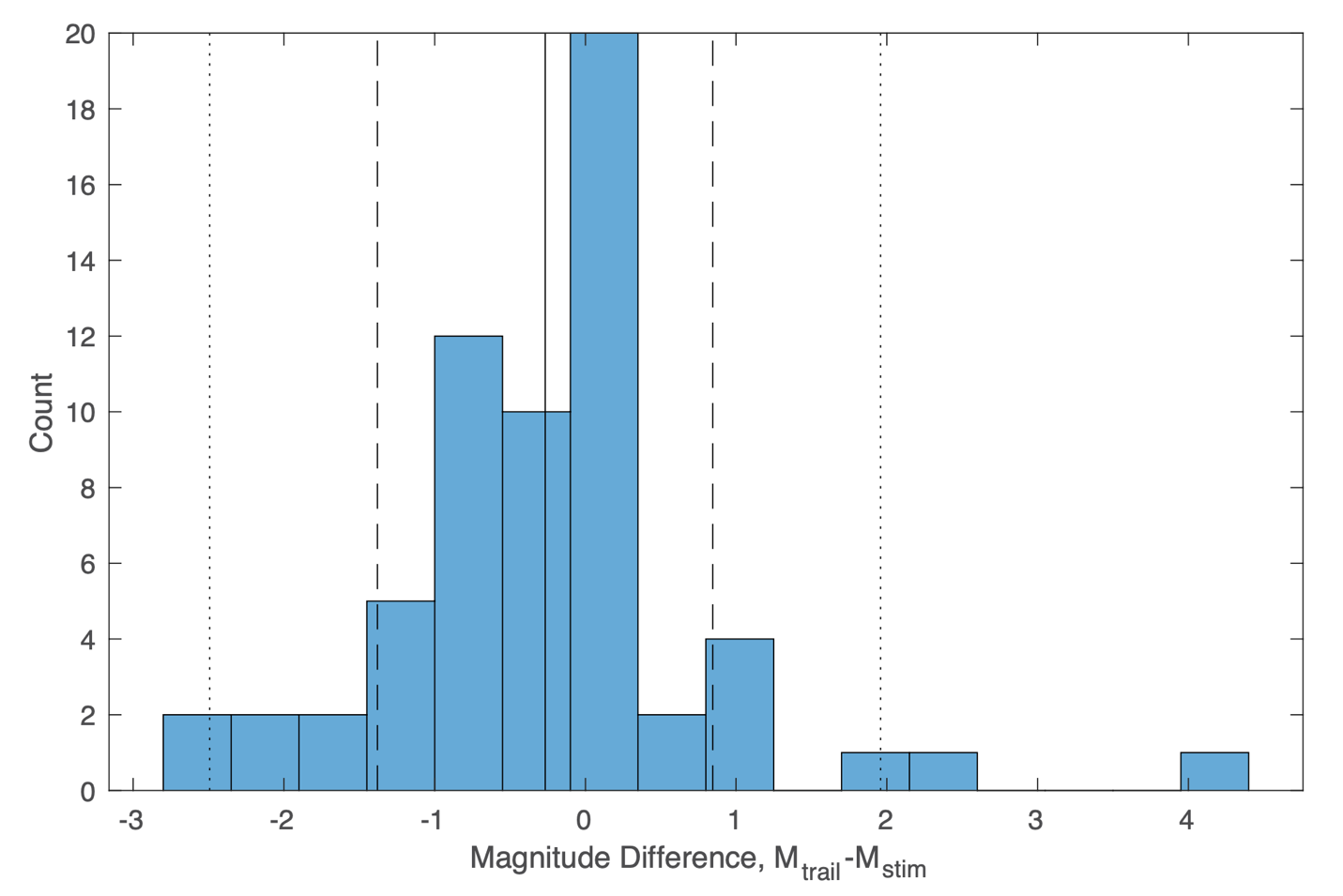


**Figure S8. Histogram of ΔM data.** Raw data histogram of ΔM data (blue bars) are shown alongside the mean (solid black line), **±**1 standard deviation (dashed black lines), and **±**2 standard deviation (dotted black lines) values of ΔM.
